# Facilitators and barriers to implementing a breastfeeding protocol for premature newborns

**DOI:** 10.1590/0034-7167-2025-0071

**Published:** 2026-05-11

**Authors:** Camila Medeiros Cruvinel Cunha, Eliane de Fátima Almeida Lima, Dulce Maria Pereira Garcia Galvão, Mônica Barros Pontes, Márcia Valéria Souza Almeida, Cândida Caniçali Primo

**Affiliations:** I Universidade Federal do Espírito Santo. Vitória, Espírito Santo, Brazil; II Escola Superior de Enfermagem de Coimbra. Coimbra, Portugal; III Hospital Universitário Cassiano Antonio Moraes. Vitória, Espírito Santo, Brazil

**Keywords:** Breast Feeding, Infant Premature, Neonatal Nursing, Implementation Science, Clinical Protocols., Lactancia Materna, Recién Nacido Prematuro, Enfermería Neonatal, Ciencia de la Implementación, Protocolos Clínicos.

## Abstract

**Objectives:**

to describe healthcare professionals’ and mothers’ perceptions regarding the facilitators/barriers to implementing a multidisciplinary breastfeeding protocol for premature and low-birth-weight newborns.

**Methods:**

participatory research, with a qualitative approach, conducted in the neonatal unit of a university hospital between June 2022 and July 2023. Sixty-two professionals and 12 mothers participated. Data were collected through an online form and workshops, and subjected to Bardin’s categorical content analysis.

**Results:**

facilitators included the presence of a multidisciplinary team, physical space for mothers to stay, family participation, and institutional documents guiding the practice. Barriers highlighted the need for improvements in hospital infrastructure, work routines, and professionals’ and family members’ commitment.

**Final Considerations:**

listening to the different stakeholders highlights the complexity of implementing evidence into clinical practice, which permeates cultural changes, work processes, relationships between people, and involves resources and service management interests.

## RESUMEN

**Objetivos:**

describir las percepciones de profesionales de la salud y madres sobre los factores que facilitan y dificultan la implementación de un protocolo multidisciplinario de lactancia materna para recién nacidos prematuros y de bajo peso al nacer.

**Métodos:**

investigación participativa, con enfoque cualitativo, realizada en la unidad neonatal de un hospital universitario entre junio de 2022 y julio de 2023. Participaron 62 profesionales y 12 madres. Los datos se recopilaron mediante formulario online y talleres, y se sometieron al análisis de contenido categórico de Bardin.

**Resultados:**

los facilitadores incluyeron la presencia de equipo multidisciplinario, espacio físico para la estancia de las madres, participación familiar y documentación institucional que orienta la práctica. Las barreras pusieron de relieve la necesidad de mejorar la infraestructura hospitalaria, rutinas de trabajo y compromiso de profesionales y familiares.

**Consideraciones Finales:**

escuchar a los diferentes actores clave pone de relieve la complejidad de implementar la evidencia en la práctica clínica, lo cual permea los cambios culturales, procesos de trabajo y relaciones interpersonales, e involucra recursos e intereses de la gestión de servicios.

## INTRODUCTION

Breastfeeding premature newborns (PTNBs) and low birth weight newborns is a complex process, requiring commitment, knowledge, and implementation of evidence-based best practices by healthcare professionals^(1)^. Mothers of PTNBs and low-birth-weight babies often face guilt, stress, and anxiety, which can directly affect breastfeeding success. Furthermore, prolonged hospitalization and the inability to breastfeed immediately after birth can be barriers to breastfeeding^(2-4)^.

Breast milk is one of the most important benefits a mother can provide to their premature baby. Studies show that continued breastfeeding in this population is associated with several benefits, such as greater weight gain, better feeding tolerance, lower infection rates, and, consequently, shorter hospital stays and reduced neonatal mortality^(5,6)^.

In this scenario, factors such as prenatal guidance, hospital approach (rooming-in and Kangaroo Method), and family and professional support in the postpartum period are extremely important for mothers’ decision to initiate and maintain breastfeeding^(7-9)^. The multidisciplinary team’s involvement, the provision of guidance to mothers by healthcare professionals, partners’ support and encouragement during breastfeeding and milk expression, and the implementation of strategies to maintain mothers’ lactation by ensuring early and frequent expression contribute to improving breastfeeding rates in neonatal units (NUs)^(10,11)^.

Furthermore, training for teams and the implementation of routines and protocols that systematize professionals’ actions and support mothers throughout the breastfeeding process are strategies that directly affect the quality of care, enabling the implementation of the best evidence for successful breastfeeding of PTNBs and low-birth-weight newborns^(1,2,7,11,12)^.

The implementation of a protocol brings benefits such as reducing variation in the care provided, directly reflecting on the quality of care and, consequently, on the execution of care to meet the specific needs of each newborn and their family^(13)^.

Although the literature presents strategies based on the best scientific evidence, implementing these strategies is a challenge for healthcare professionals^(12)^, and the hospital where this research was conducted does not yet have an evidence-based protocol for breastfeeding care for PTNBs and low-birth-weight newborns. In this context, the following guiding question emerged: can understanding the facilitators and barriers from healthcare professionals’ and mothers’ perspectives contribute to designing the necessary actions to achieve the best breastfeeding outcomes for PTNBs and low-birth-weight newborns?

### Relevance of the study

This article is a product of the master’s dissertation “Protocol for breastfeeding premature and low-birth-weight newborns,” deposited in the institutional repository (https://enfermagem.vitoria.ufes.br/pt-br/pos-graduacao/PPGENF/detalhes-de-pessoal-discente?id=671716)^(14)^.

## OBJECTIVES

To describe healthcare professionals’ and mothers’ perceptions regarding the facilitators and barriers to the implementation of a multidisciplinary breastfeeding protocol for PTNBs and low birth weight newborns.

## METHODS

### Ethical aspects

The research was approved by the Research Ethics Committee. All participants were informed of the research objective and procedures, as well as their right to refuse and withdraw from participation at any time. Informed Consent Forms were signed by all participants after agreeing to participate. The working group meetings and workshops with mothers took place in private rooms within the study institution. This research aimed to meet an institutional demand for improvements in breastfeeding care for PTNBs, and the results were presented and discussed by the multidisciplinary management committee of the institution’s NU. The entire team was informed that a project focused on breastfeeding PTNBs and low-birth-weight newborns would be initiated, and a banner was displayed highlighting the entire trajectory of the work.

### Study design and theoretical-methodological framework

This is participatory research with a qualitative approach that was developed to identify facilitators and barriers to the implementation of a best practice protocol based on the JBI Evidence Implementation Methodology^(12)^. This research report followed the criteria established in the COnsolidated criteria for REporting Qualitative research.

### Study setting and participants

The study was carried out at a university hospital in the state of Espírito Santo, with services entirely focused on users of the Brazilian Health System and a state reference for high-risk pregnancies between June 2022 and July 2023. The research unit is represented by the NU, which has 25 beds, divided between the Neonatal Intensive Care Unit (NICU), with ten beds, the Conventional Intermediate Care Unit (CoINCU), with ten beds, and the Kangaroo Intermediate Care Unit (KaINCU), with five beds.

Professionals from various categories who provide direct and indirect breastfeeding assistance to PTNBs admitted to this unit participated, including nurses, nursing technicians and assistants, physicians, physical therapists, speech therapists, an occupational therapist, a social worker, a psychologist, a nutritionist, and administrative and cleaning staff. All 132 unit employees and 24 mothers who had newborns admitted to the unit in August 2022, the month in which the workshops took place, were invited to participate in the study.

### Data collection and organization

To organize the process of implementing the clinical protocol and identifying the facilitators and barriers encountered in clinical practice in May 2022, a working group made up of different professionals was formed for convenience.

This group consisted of 14 professionals from different categories and departments of the hospital, including physicians and nurses from the management and support unit, maternity unit, and Human Milk Bank (HMB) on a permanent basis (eight people), as well as nursing technicians, a speech therapist, a social worker, an occupational therapist, a psychologist, and a nutritionist from the unit on a temporary basis. The number of participants in each meeting varied depending on the temporary members’ availability and the topic being discussed.

To identify facilitators and barriers from professionals’ perspective, an online questionnaire was applied, based on the Baby-Friendly Hospital Initiative Guide^(15)^. The instrument was reviewed by the working group for adequacy and clarity, eliminating the need for pilot testing, and was made available via institutional email for 15 days for responses. The instrument contained four professional characterization questions and two open-ended questions: “Describe, in your opinion, the facilitators for implementing a breastfeeding protocol for PTNBs” and “Describe, in your opinion, the barriers to implementing a breastfeeding protocol for PTNBs”. Sixty-two professionals, including members of the working group, responded. Since this is an online instrument, all respondents were considered, adopting the criterion of exhaustiveness rather than saturation.

To identify the facilitators and barriers mothers experience regarding breastfeeding at the unit, breastfeeding workshops were held in August 2022, in five one-hour afternoon sessions, with 12 mothers participating. The number of participants varied between three and five per session. Data collection ended when theoretical saturation was reached in the fourth workshop, and a fifth workshop was held to confirm saturation. Mothers were identified as M1, M2, M3, and M12 to preserve anonymity. Data collection with mothers ended when saturation was achieved. Data reached saturation in the fourth workshop; however, an additional workshop was held to confirm saturation due to the small number of participants per workshop.

The facilitator who led the meetings with the working group and the workshops with the mothers was the main author, a nurse at the NICU of the study institution, a master’s student in nursing, with experience in conducting qualitative research and leading group activities.

Recordings of the working group meetings and workshops held with the mothers were transcribed using a dedicated online tool (VEED.IO). Transcription was done verbatim and followed the structure of participants’ statements to maintain the accuracy of the statements. After transcription, the texts were returned to the working group for reading and analysis.

The members of the working group were responsible for analyzing the questionnaires answered by employees and mothers’ statements during the workshops, listing the challenges faced by the institution in relation to the topic, and proposing feasible solutions to improve the care provided.

Four in-person meetings with the working group were held in September, October, November, and December 2022. All meetings were recorded and later transcribed. To ensure anonymity, the professionals participating in the working group were identified as “P” in the sequence in which they spoke (P1, P2, P3, and P14).

The meetings lasted one hour and 30 minutes each and included a group assistant who kept a field diary and recorded the discussions. The assistant was a nursing assistant at the NU, a member of the hospital’s Breastfeeding Committee, established in 2022, and had experience leading groups and conducting qualitative research.

### Data analysis

From text skimming readings of the transcribed material, it was possible to extract impressions and guidance on the content to grasp, in a non-systematized manner, relevant aspects that were submitted to categorical content analysis in its three phases: 1) pre-analysis; 2) material exploration; and 3) treatment of results, inference, and interpretation^(16)^.

In pre-analysis, content spoken by interviewees unrelated to the research question was eliminated from the textual corpus. During material exploration, the research group organized the statements, grouping the content so that it could be presented appropriately. In processing the results, word classes and radicals that allowed grouping the content into meaning clusters were extracted, forming a tree of words and relationships among them^(16)^. The responses to the two open-ended questions in the instrument for healthcare professionals were grouped by frequency, and a word cloud was created using Wordcloudmaker. Three thematic categories were established, addressing the facilitators and barriers to implementing the breastfeeding protocol.

## RESULTS

Regarding the participants’ profile, healthcare professionals were between 31 and 40 years old (40.4%), with an average of 6 to 10 years of maternal and childcare experience (38.7%). The highest qualifications were at the specialization level, accounting for 58.1% of the total participants in the unit. The roles with the highest number of responses were nursing technicians (32.2%), nurses (30.6%), and physiotherapists (13%). In relation to mothers, the majority were between 19 and 35 years old (75%), and 66.7% had a partner (married/common-law relationship). Concerning education, 41.6% of women had completed high school, while 25% had incomplete high school.

In relation to professionals’ perception, the facilitators for implementing the breastfeeding protocol for PTNBs in the unit were: the presence of a complete, dedicated, and highly trained multidisciplinary team in the unit; interested and willing staff to participate in training; the presence of physical space for mothers to stay full-time; the existence of a HMB in the hospital; outpatient follow-up for PTNBs; a care plan developed together with the family and the delivery of educational materials; multidisciplinary visits; the participation of mothers; and the preparation of institutional documents that guide clinical practice, establish routines, and improve communication among the different teams.

In contrast, factors characterized as barriers to the implementation of the breastfeeding protocol were: inadequacies in infrastructure and services (the NU was located in a different building from the maternity ward, obstetric center, and HMB; there was no bathroom for bathing mothers who accompanied their children full-time; there was no place for 24-hour milk extraction and storage/collection station; and there was no place to wash and dry clothes); poor team communication; lack of awareness among professionals; lack of protocols and documents that standardize breastfeeding care; divergence of conduct among teams/professionals; some professionals felt insecure in providing guidance for establishing and maintaining breastfeeding; discontinuity in the work carried out between sectors; scarcity of breastfeeding training; lack of guidance on breastfeeding during prenatal and maternity wards; lack of in-hospital and post-discharge support groups; prolonged hospitalizations of PTNBs; lack of management support; maternal absence; and lack of parental commitment (social vulnerability).

**Figure 1:**
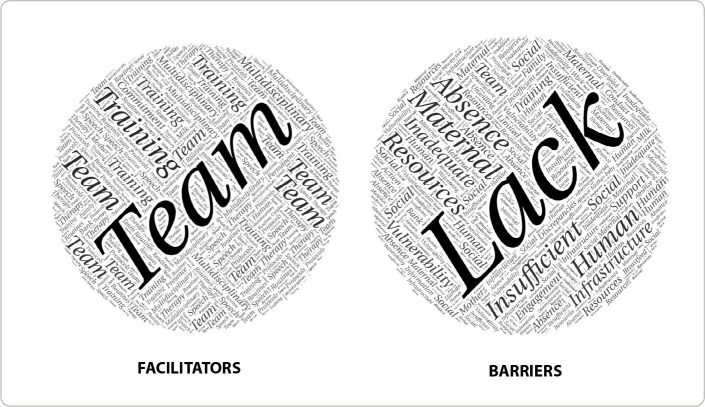
Text corpus analysis of facilitators (left) and barriers (right), as perceived by staff, for implementing a breastfeeding routine in the neonatal unit

Figure 1 shows it is possible to visualize, from the employees’ perspective, the facilitators and barriers to implementing a premature breastfeeding protocol in the NU, represented by a word cloud.

In mothers’ perception, facilitators were the presence of a multidisciplinary team and staff from the maternity ward, NU, and HMB who were interested in helping and guiding. Meanwhile, barriers were related to the department’s infrastructure and routine. Mothers cited the lack of a bathroom near the unit and the poor quality of available chairs as barriers, factors that limited their stay in the department. Furthermore, many reported having a cesarean section and experiencing pain, making it difficult to travel between buildings. In this context, mothers must travel from one floor to another (sometimes by stairs) to use the bathroom or from one building to another to shower and go to the HMB.

Mothers reported that chairs are low and of poor quality, making it difficult for them to sit or stand up on their own, remain seated for long periods, or breastfeed for long periods. They also noted that there is no place to wash and dry their clothes in the NU building, nor elsewhere in the hospital, making personal hygiene difficult.

Mothers also reported issues related to feeding, such as limited mealtimes and difficulty accessing the cafeteria. They highlighted long lines, long waits for meals, and the lack of covered routes, which are physically exhausting for mothers. Furthermore, mealtimes are scheduled, often coinciding with feeding times. This makes it difficult for mothers to go to the cafeteria, as breastfeeding hours can be extended, including breast massage, milk extraction, and breastfeeding for PTNBs learning to suckle.

Based on the content analysis of professionals’ and mothers’ perceptions, it was observed that the facilitators and barriers are related to three major thematic categories: Facilitators and barriers related to infrastructure and intersectoral processes; Facilitators and barriers related to the routines and care of healthcare professionals; and Facilitators and barriers related to mothers and family members.

### Facilitators and barriers related to infrastructure and intersectoral processes

In this category, facilitators included the presence of physical space for mothers to stay full-time, the existence of an HMB in the hospital, and outpatient follow-up for PTNBs. On the other hand, barriers identified were: the NU was located in a different building from the maternity ward, obstetric center, and HMB; the lack of a bathroom in the NU building; poor-quality chairs; the lack of a place to wash and dry clothes; difficult access to the cafeteria; limited meal times that coincide with feeding times; and the lack of a place to express and store milk 24 hours a day.

*The institution needs to offer adequate structure and support for the mother to remain in the service.* (P13)

*In the NICU, all mothers must be referred to the Milk Bank, regardless of whether they have difficulties or not.* (P7)

*Bathroom on the same floor as CoINCU and KaINCU: this is a problem. We only have one bathroom with a toilet. Bathrooms with showers are only in the maternity ward, going up and down to the maternity ward, having surgery, getting there, and having to wait to use the bathroom with two or three other patients is unfeasible.* (P13)

*The bathroom could be closer for mothers with babies in the NICU.* (M4)

*The nursing chairs are bad.* (M5)

*It takes a while to get to eat, and sometimes we have to go in the rain or shine. Not to mention the times we get to the cafeteria, and it’s already closed.* (M11)

*Lunch is at the time of bedside milking and feeding, and the breastfeeding process for these babies is slow. Mothers often also don’t have access to afternoon tea because that’s when they’re breastfeeding again.* (P16)

*We have a serious problem at weekends. Mom has a lot of milk, and we don’t have a refrigerator to store the milk she expresses.* (P2)

*We often throw away milk, and the mother feels frustrated.* (P11)

### Facilitators and barriers related to the routines and care of healthcare professionals

Most facilitators were identified in this category, namely: having a complete, dedicated, and highly trained multidisciplinary team in the unit; interested and willing employees to participate in training; implementing a care plan developed together with the family and delivering educational materials; conducting multidisciplinary visits; and preparing institutional documents to improve team routines and communication.

On the other hand, professionals and mothers pointed out: barriers related to communication within the team; lack of protocols and documents that standardize breastfeeding care; divergence of conduct among teams/professionals; feelings of insecurity among some professionals in guiding for establishing and maintaining breastfeeding; lack of breastfeeding training in the hospital; lack of awareness among professionals to welcome and support families; discontinuity in the work carried out between departments; lack of guidance on breastfeeding during prenatal and maternity care; prolonged hospitalizations of infants; and lack of in-hospital and post-discharge support groups.

*This working group represents the interests of professionals in supporting and promoting breastfeeding in the hospital.* (P1)

*Everything we do is guided by literature, but we have to adapt it to our reality.* (P14)

*We identify a need for better communication. The NICU and the HMB need to communicate better.* (P9)

*We need to create an instrument that is formalized... when the unit does not have a defined protocol for a practice, it is jointly responsible for the failure.* (P6)

*All guidance must begin during prenatal care.* (P10)

*The improvement in breastfeeding rates is directly associated with the fact that this guidance begins in the maternity ward.* (P13)

*This impact on breastfeeding comes along with the issue of establishing the third stage, in which the patient is discharged and has no support.* (P8)

### Facilitators and barriers related to mothers and family members

Healthcare professionals highlighted the presence of mothers as a facilitator. However, barriers included maternal absence, a highly socially vulnerable population, and the difficulty and delay in including fathers in childcare.

*The exception is the mother who stays, so we have to work a lot with her.* (P2)

*While the baby is in the NICU, this mother visits sporadically. How will we maintain her milk supply when the baby is ready for oral feeding, since she’s not in the unit? Ultimately, the baby is ready for discharge, but breastfeeding hasn’t happened. We need to find a way to work with them upon this baby’s arrival in the NICU.* (P12)

*It’s important to consider that these mothers have undergone surgery, ride the bus, live in the hills, and leave within 48 hours, only to have to return to spend a full day in the hospital. They have small children; they face a whole social issue.* (P3)

*The mother has returned home. It seems like household chores are overwhelming her. Not to mention that the first 72 hours are crucial for working with this mother, still in the cradle, to create a routine and demystify some things. Helping the mother understand her role in relation to her child.* (P10)

*Motherhood and fatherhood happen after birth, not during pregnancy. If we interrupt this bond after birth, reestablishing it is much more difficult, and this causes parents to become increasingly distant and end up believing that those providing care take better care of their child than they do.* (P16)

*This impacts hospital discharge. The flow of care for mothers and fathers in caring for their babies is occurring late in the NICU.* (P15)

*It is important to consider that having the mother nearby reduces the length of the newborn’s hospital stay.* (P12)

Among the various barriers encountered, the working group selected 11 to be addressed in the best evidence implementation project. These barriers were chosen based on their relevance and feasibility, as identified by the working group’s professionals. The participation of professionals and mothers allowed for the identification of barriers in different areas. During the months of discussions, the 11 selected barriers were adjusted based on strategies developed with healthcare professionals, family members, and management, which enabled the implementation of the institution’s first breastfeeding protocol for PTNBs^(17)^. It is worth noting that the protocol was built based on the best scientific evidence and following the institution’s standard for improving care; therefore, its focus was directly related to overcoming the barriers encountered, without addressing facilitators, topics in which the team had already demonstrated success.

## DISCUSSION

About the facilitators and barriers related to infrastructure and intersectoral processes, it is worth reflecting that, despite the existence of legislation defining guidelines for the organization of comprehensive and humanized care for newborns admitted to NUs, many places in Brazil still have limitations in the routine and physical structure offered to parents and employees^(18)^. Research carried out in 606 maternity hospitals in 408 Brazilian municipalities revealed a lack of hospital infrastructure and a lack of privacy related to the meals offered, as well as inadequate facilities for bathing, resting, and a lack of bed linen^(19)^.

Other studies have also identified barriers to breastfeeding premature babies as: deficient infrastructure at the NICU; distance between the NICU and the mother’s room; lack of routine for healthcare professionals; inadequate bathrooms; furniture such as broken chairs and insufficient quantity; lack of a place to bathe and rest; and lack of laundry facilities for washing and drying clothes for mothers accompanying their babies^(20-22)^.

Regarding the lack of a place to express and store milk, it is important to note that the hospital’s HMB is not open 24 hours a day or on weekends, and therefore, a collection point or storage location for expressed milk would be important. The HMB plays an important role in establishing breastfeeding for PTNBs during their hospitalization, aiding in the weight gain of PTNBs receiving pasteurized or raw human milk, and supporting mothers in maintaining lactation and expressing breast milk^(23)^.

It is known that breast milk has essential immunological properties to aid in the gastrointestinal maturation of premature babies, in the prevention of infections, in better neurobehavioral performance, and in the mother-child bond^(1,7)^. Breast pumping should begin as early as possible and continue at least eight times a day for mothers who are not yet breastfeeding, ensuring milk production maintenance. Professionals must modify their hospital practices, adopting early breast pumping and breast milk supply for PTNBs^(2,9,10)^.

Concerning facilitators and barriers related to routines and care provided by healthcare professionals, research has identified guidance and support from staff, training, and an adequate number of professionals in the NICU as facilitators of breastfeeding premature babies^(20)^. Meanwhile, the barriers encountered were a lack of encouragement and guidance for mothers; difficulty in communication between healthcare professionals and mothers; lack of preparation of mothers in prenatal care; and lack of humanization of care^(21)^.

Effective communication is crucial from the mother’s first contact with the neonatal team. It is through this process that trust and partnership are built for prolonged hospitalizations, lasting from weeks to months. It is also important to note that ineffective communication within the team and between departments leads to conflicting guidelines, inconsistent information, omission or transmission of erroneous information, and illegible records, all of which impact patient safety^(1,24)^.

As for breastfeeding guidance, initiating ongoing breastfeeding education early during prenatal care strengthens and prepares family members for newborn’s arrival. It is also important to address pregnant women before delivery and during hospitalization in the maternity ward as decision-makers for a more guided and peaceful hospitalization^(1-3,21)^.

Mothers emphasize the importance of receiving information and support from professionals in the first few days after birth when questions and difficulties arise. During this period, they are still hospitalized and surrounded by professionals trained to offer this service^(22)^.

The need to form in-hospital and post-discharge support groups is highlighted to strengthen the bond between the two, clarify the most frequent doubts, and promote safer discharges^(1,2)^. Social network support is essential for exclusive breastfeeding, sustaining the mothers’ presence in the unit, and reducing newborn hospitalization time and women’s burden^(4,25)^.

The main reasons reported by mothers for total or partial weaning after discharge are related to cultural and educational issues, requiring guidance regarding milk production and the breastfeeding process in order to reduce the perception of low milk production^(8)^.

Regarding facilitators and barriers related to mothers and family members, a study showed that, according to NICU healthcare professionals, the facilitators of breastfeeding premature babies are: constant presence of the mother with free access to the unit; Kangaroo Method with skin-to-skin contact; mother-baby interaction and bonding; early initiation and stimulation; newborn conditions; and mothers’ desire^(20)^. On the other hand, maternal unavailability, low milk production, mother-baby separation, maternal social issues, and prolonged hospitalization time of PTNBs were factors that made breastfeeding difficult for PTNBs^(21)^.

Interventions that improve breastfeeding outcomes include policies such as the Baby-Friendly Hospital Initiative and the Kangaroo Method, as well as the importance of continuity of care and support in community and family settings, through home visits, with the involvement of parents, grandparents, and the community^(25)^.

The guidance, encouragement, and support that women receive from family, friends, and healthcare professionals are essential for them to feel welcomed, comfortable, and capable of breastfeeding^(22)^.

It is important to note that most breastfeeding challenges are related to structural issues, not to factors or even to women’s personal and individual decisions. Ensuring respectful, culturally appropriate, non-stigmatizing, and evidence-based care is essential to achieving breastfeeding for PTNBs and low-birth-weight newborns^(1,2)^.

### Study limitations

One limitation of this study is the limited number of professionals and mothers who participated, which may have limited the diversity of perspectives considered. The need to establish institutional strategies that foster greater engagement and adherence among different stakeholders in the proposed activities is emphasized, considering the relevance of these actions in strengthening the bond between families and the healthcare team. Furthermore, the findings represent the perceptions of a specific group of participants involved in a local experience, which limits the possibility of generalizing the results to other institutions and healthcare settings.

### Contributions to nursing and health

The study contributes to nursing by enabling reflections on the convergences and contrasts related to breastfeeding care in a NU. The research warns that maintaining evidence-based care requires ongoing education of healthcare professionals, as well as active listening and establishing partnerships with parents and support networks for breastfeeding initiation and maintenance for PTNBs. Nurses need to understand that facilitators and barriers reflect the complexity of implementing best evidence into clinical practice, which permeates cultural changes, work processes, relationships between people, and involves resources and service management interests.

## FINAL CONSIDERATIONS

This research described healthcare professionals’ and mothers’ perceptions regarding the facilitators and barriers to implementing a breastfeeding protocol for PTNBs, highlighting the importance of listening to the actors involved in the process to implement evidence-based best practices and ensure exclusive breastfeeding of PTNBs.

The formation of a multidisciplinary working group allowed all categories to be heard, improving communication within the department and the institution. Furthermore, workshops with mothers allowed them to better connect with the service and be more open to discussing issues inherent to their children’s hospitalization and the challenges faced in the unit regarding breastfeeding guidance and maintenance.

The presence of a comprehensive and dedicated multidisciplinary team at the NU, and the availability of services that serve as pillars of PTNB care (such as the HMB and outpatient follow-up for PTNBs), combined with the creation of clinical practice guidance documents, were identified as facilitators for implementing a breastfeeding routine. Conversely, barriers can be represented by poor team communication, structural and service inadequacies, difficult relationships with other hospital departments, lack of management support, and issues that go beyond professionals’ governance, represented by the high social vulnerability of the population assisted.

In general, from healthcare professionals’ and mothers’ perspectives, mentions of the need for improvements in hospital infrastructure, work routines, and processes, and the commitment of professionals and family members are noteworthy.

Several challenges remain to be overcome. However, this research enabled the identification of service needs, with a view to improving the quality of care provided and promoting holistic care, focused on addressing these families’ physical and emotional health.

## Data Availability

The research data are available within the article.
