# Supplementary material for: Facilitators and barriers to implementing a breastfeeding protocol for premature newborns
Source: Rev Bras Enferm. 2026 May 11;79:e20250071. doi: 10.1590/0034-7167-2025-0071 (PMC13169627; doi:10.1590/0034-7167-2025-0071)
Supplement: Translation

# Translation: Facilitadores e barreiras na implantação de protocolo de amamentação para prematuros

**DOI:** 10.1590/0034-7167-2025-0071pt

**Authors:** Camila Medeiros Cruvinel Cunha, Eliane de Fátima Almeida Lima, Dulce Maria Pereira Garcia Galvão, Mônica Barros Pontes, Márcia Valéria Souza Almeida, Cândida Caniçali Primo

**Affiliations:** I Universidade Federal do Espírito Santo. Vitória, Espírito Santo, Brasil; II Escola Superior de Enfermagem de Coimbra. Coimbra, Portugal; III Hospital Universitário Cassiano Antonio Moraes. Vitória, Espírito Santo, Brasil

**Keywords:** Amamentação, Recém-Nascido Prematuro, Enfermagem Neonatal, Ciência da Implementação, Protocolos Clínicos.

## Abstract

**Objetivos:**

descrever a percepção dos profissionais de saúde e mães acerca dos facilitadores/barreiras para implantação de protocolo multidisciplinar de amamentação para recém-nascido prematuro e de baixo peso.

**Métodos:**

pesquisa participativa, com abordagem qualitativa, desenvolvida na unidade neonatal de hospital universitário entre junho de 2022 e julho de 2023. Participaram 62 profissionais e 12 mães. Os dados foram coletados por meio de formulário *online* e oficinas, submetidos à análise de conteúdo categorial de Bardin.

**Resultados:**

os facilitadores incluíram presença de equipe multiprofissional, espaço físico para permanência das mães, participação da família e documentos institucionais norteadores da prática. As barreiras apontaram necessidade de melhoria na infraestrutura do hospital, nas rotinas de trabalho e no comprometimento dos profissionais e familiares.

**Considerações Finais:**

a escuta dos diferentes atores evidencia a complexidade da implementação de evidências na prática clínica, que perpassa mudanças culturais, processos de trabalho, relações entre pessoas, envolvendo recursos e interesses da gestão dos serviços.

## INTRODUÇÃO

A amamentação de recém-nascidos prematuros (RNPTs) e de baixo peso é um processo complexo, requerendo compromisso, conhecimento e implementação das melhores práticas baseadas em evidências por parte dos profissionais de saúde^(1)^. As mães de RNPTs e de baixo peso muitas vezes enfrentam culpa, estresse e ansiedade, o que pode afetar diretamente o sucesso da amamentação. Além disso, a vivência da hospitalização prolongada e a impossibilidade de amamentar logo após o nascimento se mostram como barreiras nesse processo^(2-4)^.

O leite materno é um dos benefícios mais importantes que uma mãe pode dar ao seu RNPT. Estudos mostram que a manutenção da amamentação nesse público está associada a diversos benefícios, como maior ganho ponderal, melhor tolerância alimentar, menores taxas de infecções e, consequentemente, menor tempo de internação e redução da mortalidade neonatal^(5,6)^.

Nesse cenário, fatores como orientações pré-natais, abordagem hospitalar (alojamento conjunto e Método Canguru), e apoio familiar e dos profissionais no pós-parto são de extrema importância para a decisão da mãe de iniciar e manter a amamentação^(7-9)^. O envolvimento da equipe multidisciplinar, o fornecimento de orientação às mães pelos profissionais de saúde, o apoio e encorajamento do parceiro durante a amamentação e expressão do leite, e a implantação de estratégias para manutenção da lactação das mães garantindo ordenha precoce e frequente contribuem para melhorar as taxas de amamentação em unidades neonatais (UNs)^(10,11)^.

Ainda, treinamentos para as equipes, e implantação de rotinas e protocolos que sistematizem as ações dos profissionais e apoiem as mães durante todo o processo da amamentação são estratégias que afetam diretamente a qualidade da assistência, possibilitando a implantação das melhores evidências para o sucesso da amamentação de RNPT e de baixo peso^(1,2,7,11,12)^.

A implementação de um protocolo traz como benefícios a redução da variação do atendimento prestado, refletindo diretamente na qualidade da assistência e, consequentemente, na execução de cuidados para atender às necessidades específicas de cada recém-nascido e sua família^(13)^.

Embora a literatura apresente as estratégias baseadas nas melhores evidências científicas, implantar essas estratégias é um desafio para os profissionais de saúde^(12)^, e o hospital cenário desta pesquisa ainda não possui um protocolo baseado em evidências para assistência à amamentação de RNPT e recém-nascido de baixo peso. Nesse contexto, emergiu a seguinte pergunta norteadora: conhecer os facilitadores e barreiras a partir dos olhares dos profissionais de saúde e mães pode contribuir para desenhar as ações necessárias para alcançar os melhores resultados na amamentação de RNPT e recém-nascido de baixo peso?

### Relevância do estudo

Este artigo é produto da dissertação de mestrado “Protocolo para aleitamento materno do recém-nascido prematuro e de baixo peso”, depositada no repositório institucional (https://enfermagem.vitoria.ufes.br/pt-br/pos-graduacao/PPGENF/detalhes-de-pessoal-discente?id=671716)^(14)^.

## OBJETIVOS

Descrever a percepção dos profissionais de saúde e mães acerca dos facilitadores e barreiras para a implantação de protocolo multidisciplinar de amamentação para RNPT e recém-nascido de baixo peso.

## MÉTODOS

### Aspectos éticos

A pesquisa foi aprovada no Comitê de Ética em Pesquisa. Todos os participantes foram informados sobre o objetivo e os procedimentos a serem realizados na pesquisa, bem como de seus direitos de recusa e de desistir de participar a qualquer momento. O Termo de Consentimento Livre e Esclarecido foi assinado por todos os participantes após aceitarem participar da pesquisa. Os encontros com o grupo de trabalho e as oficinas com as mães aconteceram em salas privadas, dentro da instituição de estudo. Esta pesquisa visou atender a uma demanda institucional por melhorias na assistência à amamentação dos prematuros, e os resultados foram apresentados e discutidos no colegiado gestor multidisciplinar da unidade neonatal da instituição. Toda a equipe foi informada de que seria iniciado um projeto voltado à amamentação de RNPTs e de recém-nascidos de baixo peso, com a fixação de um *banner* que expôs toda a trajetória do trabalho.

### Tipo de estudo e referencial teórico-metodológico

Pesquisa participativa, com abordagem qualitativa, desenvolvida para identificar facilitadores e barreiras na implementação de protocolo de melhores práticas baseado na Metodologia de Implementação de Evidências do JBI^(12)^. Este relatório de pesquisa seguiu os critérios estabelecidos no *COnsolidated criteria for REporting Qualitative research*.

### Cenário e participantes de estudo

O estudo foi realizado em um hospital universitário no estado do Espírito Santo com atendimento totalmente voltado aos usuários do Sistema Único de Saúde e referência estadual em gestação de alto risco entre junho de 2022 e julho de 2023. A unidade de investigação é representada pela UN, que conta com 25 leitos, divididos entre Unidade de Terapia Intensiva Neonatal (UTIN), com dez leitos, Unidade de Cuidados Intermediários Convencional (UCINCo), com dez leitos, e Unidade de Cuidados Intermediários Canguru (UCINCa), com cinco leitos.

Participaram profissionais de diversas categorias que prestam assistência direta e indireta na amamentação de RNPT internados nessa unidade, representados por enfermeiros, técnicos e auxiliares de enfermagem, médicos, fisioterapeutas, fonoaudiólogas, terapeuta ocupacional, assistente social, psicóloga, nutricionista, e colaboradores do administrativo e do serviço de higienização. Foram convidados para participar do estudo todos os 132 funcionários da unidade e as 24 mães que tinham seu recém-nascido internado na unidade no mês de agosto de 2022, mês em que ocorreram as oficinas.

### Coleta e organização dos dados

Para a organização do processo de implementação do protocolo clínico e identificação dos facilitadores e das barreiras encontradas na prática clínica em maio de 2022, foi constituído, por conveniência, um grupo de trabalho formado por diferentes profissionais.

Este grupo foi composto por 14 profissionais de diferentes categorias e setores do hospital, como médicas e enfermeiras gerenciais e assistenciais da UN, maternidade e Banco de Leite Humano (BLH) de forma fixa (oito pessoas), além de técnicos de enfermagem, fonoaudióloga, assistente social, terapeuta ocupacional, psicóloga e nutricionista da UN de maneira flutuante. O número de participantes em cada encontro variou conforme a disponibilidade dos membros flutuantes e a pauta abordada.

Para identificar facilitadores e barreiras sob a perspectiva dos profissionais, foi aplicado um questionário *online* elaborado a partir do Guia da Iniciativa Hospital Amigo da Criança^(15)^. O instrumento foi revisado pelo grupo de trabalho para adequação e clareza, dispensando a necessidade de teste piloto, e foi disponibilizado via e-mail institucional por 15 dias para resposta. O instrumento continha quatro questões de caracterização profissional e duas questões abertas: “Descreva, na sua opinião, quais os facilitadores para implantação de um protocolo de amamentação para RNPTs” e “Descreva, na sua opinião, quais as barreiras para implantação de um protocolo de amamentação para RNPTs”. Responderam 62 profissionais, incluindo membros do grupo de trabalho. Por se tratar de instrumento *online*, considerou-se a totalidade dos respondentes, adotando o critério de exaustividade e não de saturação.

Para identificar os facilitadores e barreiras vivenciados pelas mães com relação à amamentação na unidade, foram realizadas oficinas sobre amamentação em agosto de 2022, em cinco encontros, com duração de uma hora cada, no período vespertino, com participação de 12 mães. O número de participantes variou entre três e cinco por encontro. A coleta foi encerrada quando atingida a saturação teórica na quarta oficina, sendo realizada uma quinta oficina para confirmação da saturação. As mães foram identificadas como M1, M2, M3... M12, para preservar o anonimato. A coleta de dados com as mães foi encerrada quando houve saturação das respostas. Os dados foram saturados na quarta oficina; no entanto, realizou-se mais uma oficina para confirmação da saturação devido ao pequeno número de participantes por oficina.

A facilitadora que conduziu os encontros com o grupo de trabalho e as oficinas com as mães foi a autora principal, enfermeira da UTIN da instituição de estudo, mestranda em enfermagem, com experiência na realização de pesquisa qualitativa e na condução de atividades em grupo.

As gravações dos encontros dos grupos de trabalho e das oficinas realizadas com as mães foram transcritas por ferramenta *online* específica (VEED.IO). A transcrição foi realizada na íntegra e obedeceu à estrutura das falas dos participantes para manter a fidedignidade dos depoimentos. Após transcrição, os textos foram devolvidos ao grupo de trabalho para leitura e análise.

Os membros do grupo de trabalho tiveram como função realizar a análise dos questionários respondidos pelos funcionários e dos depoimentos das mães durante as oficinas, elencando os desafios enfrentados na instituição com relação ao tema, propondo soluções factíveis para melhoria do cuidado prestado.

Foram realizados quatro encontros presenciais com o grupo de trabalho, ocorridos nos meses de setembro, outubro, novembro e dezembro de 2022. Todos os encontros foram gravados e posteriormente transcritos. Para assegurar o anonimato, os profissionais participantes do grupo de trabalho foram identificados como “P” na sequência em que se manifestavam (P1, P2, P3... P14).

Os encontros tiveram duração de uma hora e 30 minutos cada, e contaram com uma auxiliar do grupo para realização do diário de campo e gravação das discussões. A auxiliar era enfermeira assistencial da UN, fazia parte da Comissão de Aleitamento Materno do hospital, estabelecida em 2022, e tinha experiência na condução de grupos e pesquisa qualitativa.

### Análise dos dados

A partir de leituras flutuantes do material transcrito, foi possível extrair impressões e orientações sobre o conteúdo para apreender, de maneira não sistematizada, aspectos relevantes que foram submetidos à análise de conteúdo categorial em suas três fases: 1) pré-análise; 2) exploração do material; e 3) tratamento dos resultados, inferência e interpretação^(16)^.

Na pré-análise, o conteúdo dito pelos entrevistados não relacionado à pergunta da pesquisa foi eliminado do corpus textual. Na exploração do material, os enunciados foram ordenados pelo grupo de pesquisa, agrupando os conteúdos para que fossem apresentados de forma adequada. No tratamento dos resultados, as classes de palavras e os radicais que permitissem agrupar os conteúdos em núcleos de sentidos foram extraídos, formando a árvore de palavras e relações entre elas^(16)^. Ainda as respostas às duas questões abertas no instrumento para os profissionais de saúde foram agrupadas pela frequência, sendo elaborada uma nuvem de palavras no *software Wordcloudmaker*. Ao final, foram estabelecidas três categorias temáticas, que abordavam os facilitadores e as barreiras para implantação do protocolo de amamentação.

## RESULTADOS

Quanto ao perfil dos participantes, os profissionais de saúde apresentaram idade entre 31 e 40 anos (40,4%), com uma média de experiência materno-infantil entre 6 e 10 anos (38,7%). A titulação máxima se concentrou no nível de especialização, representando 58,1% do total de participantes da unidade. As funções com maior número de respostas foram representadas por técnicos de enfermagem (32,2%), enfermeiros (30,6%) e fisioterapeutas (13%). Em relação às mães, a maioria apresentava idade entre 19 e 35 anos (75%), e 66,7% possuíam parceiro (casada/união estável). Em relação à escolaridade, 41,6% das mulheres possuíam ensino médio completo, enquanto 25% possuíam ensino médio incompleto.

Com relação à percepção dos profissionais, os facilitadores para implantação do protocolo de amamentação para RNPT na unidade foram: presença na unidade de equipe multiprofissional completa, exclusiva e com alto grau de formação; funcionários interessados e dispostos a participar de treinamentos; presença de espaço físico para permanência das mães em tempo integral; existência de BLH no hospital; seguimento ambulatorial para prematuros; plano de cuidados construído junto com a família e entrega de material educativo; realização de visita multiprofissional; participação das mães; e elaboração de documentos institucionais que norteiam a prática clínica, estabelecem rotinas e melhoram a comunicação entre as diferentes equipes.

Em contrapartida, fatores caracterizados como barreiras à implantação do protocolo de amamentação foram: inadequações na infraestrutura e nos serviços (UN situada em prédio diferente da maternidade, centro obstétrico e BLH, ausência de banheiro para banho das mães que acompanham seus filhos em tempo integral, ausência de local para extração e armazenamento de leite 24 horas/posto de coleta, e ausência de local para lavar e secar roupas); comunicação deficiente da equipe; falta de sensibilização dos profissionais; ausência de protocolo e documentos que padronizem a assistência em amamentação; divergência de condutas entre equipes/profissionais; sentimento de insegurança de alguns profissionais para fornecer orientações para estabelecimento e manutenção da amamentação; descontinuidade no trabalho realizado entre os setores; escassez de treinamentos sobre amamentação; falta de orientação no pré-natal e na maternidade sobre amamentação; inexistência de grupos de apoio intra-hospitalares e no pós-alta; internações prolongadas dos RNPT; falta de apoio da gestão; ausência materna; e falta de comprometimento dos pais (vulnerabilidade social).

Na Figura 1, é possível visualizar, na percepção dos funcionários, os facilitadores e as barreiras para implantação de protocolo de amamentação de prematuros na UN, representados por meio de nuvem de palavras.

**Figura 1:** Análise de *corpus* textual acerca dos facilitadores (a esquerda) e das barreiras (a direita), na percepção de funcionários, para implantação de rotina de aleitamento materno na unidade neonatal

Na percepção das mães, os facilitadores foram a presença de uma equipe multiprofissional e de funcionários da maternidade, UN e BLH estarem interessados em ajudar e orientar. Enquanto isso, as barreiras estiveram relacionadas à infraestrutura e rotina do setor. As mães apontaram como barreiras falta de um banheiro próximo à unidade e a má qualidade das cadeiras disponíveis, fatores que limitavam sua permanência no setor. Além disso, muitas relataram que tiveram parto cesárea e que sentiam dor, sendo difícil fazer o deslocamento entre os prédios. Ainda nesse contexto, as mães precisam se deslocar de um andar para outro (algumas vezes por escadas) para utilizar o banheiro ou de um prédio para outro para tomar banho e ir ao BLH.

As mães informaram que as cadeiras são baixas e de má qualidade, dificultando que elas se sintam ou se levantem sozinhas, ou que permaneçam sentadas por muito tempo ou que amamentem por longos períodos. Também apontaram não existir no prédio da UN, nem em outro lugar do hospital, local para lavar e secar suas roupas, dificultando os cuidados de higiene pessoal.

Também foram relatadas pelas mães questões relacionadas à alimentação, como horários limitados de refeição e dificuldade de acesso ao refeitório, ressaltando-se as extensas filas, a necessidade de aguardar muito tempo para comer e o trajeto não ser coberto, favorecendo o desgaste físico dessa mãe. Além disso, as refeições ocorrem em horários pré-determinados, muitas vezes coincidentes com os horários de mamada. Essa questão dificulta a ida das mães ao refeitório, visto que o horário de amamentação pode se estender, considerando o processo desde a massagem das mamas, extração de leite e amamentação dos RNPTs que estão aprendendo a sugar.

A partir da análise de conteúdo das percepções dos profissionais e das mães, observou-se que os facilitadores e as barreiras estão relacionados a três grandes categorias temáticas: Facilitadores e barreiras relacionados à infraestrutura e aos processos intersetoriais; Facilitadores e barreiras relacionados às rotinas e aos cuidados dos profissionais de saúde; e Facilitadores e barreiras relacionados às mães e familiares.

### Facilitadores e barreiras relacionados à infraestrutura e aos processos intersetoriais

Nesta categoria, verificou-se como facilitadores a presença de espaço físico para permanência das mães em tempo integral, a existência de BLH no hospital e o seguimento ambulatorial para prematuros. Por outro lado, as barreiras identificadas foram: UN localizada em prédio diferente da maternidade, centro obstétrico e BLH; ausência de banheiro para banho no prédio da UN; baixa qualidade das cadeiras; ausência de local para lavar e secar roupas; dificuldade de acesso ao refeitório; horários de refeição limitados e coincidentes com os horários de mamada; e ausência de local para extração e armazenamento de leite 24 horas.

*Há necessidade de a instituição oferecer estrutura e suporte adequados para a mãe permanecer no serviço.* (P13)

*Na UTIN, todas as mães devem ser encaminhadas ao Banco de Leite, independentemente de terem dificuldade ou não.* (P7)

*Banheiro no mesmo andar da UCINCo e UCINCa: esse é um problema. Só temos um banheiro, com um vaso sanitário. Banheiro com chuveiro, só na maternidade... descer e subir até a maternidade, operada, chegar lá e ficar esperando para usar o banheiro com mais duas ou três pacientes é inviável.* (P13)

*O banheiro podia ser mais próximo para as mães com bebês na UTIN*. (M4)

*As cadeiras para amamentar são ruins.* (M5)

*A gente demora para ir comer e às vezes ainda tem que ir à chuva ou no sol. Sem falar das vezes que chegamos ao refeitório e já está fechado.* (M11)

*O almoço é no horário da ordenha a beira-leito e mamada, e o processo de amamentação desses bebês é demorado. Muitas vezes, as mães também não têm acesso ao café da tarde, porque é o horário em que elas estão amamentando novamente.* (P16)

*Nós temos um problema sério nos finais de semana. Mãe cheia de leite e nós não temos uma geladeira para guardar o leite que ela tira.* (P2)

*Muitas vezes, jogamos leite fora e a mãe se sente frustrada.* (P11)

### Facilitadores e barreiras relacionados às rotinas e aos cuidados dos profissionais de saúde

A maioria dos facilitadores foi apontada nesta categoria, sendo eles: possuir equipe multiprofissional completa, exclusiva e com alto grau de formação na unidade; funcionários interessados e dispostos a participar de treinamentos; realizar plano de cuidados construído junto com a família e entregar material educativo; realizar visita multiprofissional; e elaborar documentos institucionais para melhorar as rotinas e a comunicação das equipes.

Por outro lado, profissionais e mães apontaram: barreiras relacionadas à comunicação dentro da equipe; ausência de protocolo e documentos que padronizem a assistência em amamentação; divergência de condutas entre equipes/profissionais; sentimento de insegurança de alguns profissionais para fornecer orientações para estabelecimento e manutenção da amamentação; escassez de treinamentos sobre amamentação no hospital; falta de sensibilização dos profissionais para acolher e apoiar as famílias; descontinuidade no trabalho realizado entre os setores; falta de orientação no pré-natal e na maternidade sobre amamentação; internações prolongadas dos lactentes; e ausência de grupos de apoio intra-hospitalares e no pós-alta.

*Esse grupo de trabalho representa o interesse de profissionais no apoio e na promoção à amamentação no hospital.* (P1)

*Tudo o que fazemos é pautado na literatura, só que temos que adaptar à nossa realidade.* (P14)

*A gente identifica uma necessidade de melhor se comunicar. UTIN e BLH precisam se comunicar melhor.* (P9)

*Precisamos criar um instrumento que seja formalizado... quando a unidade não tem um protocolo definido sobre uma prática, ela é corresponsável pela falha.* (P6)

*Todas as orientações têm que começar no pré-natal.* (P10)

*A melhoria das taxas de aleitamento está diretamente associada ao fato de essa orientação começar na maternidade.* (P13)

*Esse impacto na amamentação vem junto com a questão do estabelecimento da terceira etapa, em que o paciente vai de alta e não tem a retaguarda.* (P8)

### Facilitadores e barreiras relacionados às mães e familiares

Os profissionais de saúde apontaram como facilitador a participação das mães quando presentes. Por outro lado, as barreiras foram ausência materna, público com grande vulnerabilidade social, e dificuldade e demora em inserir os pais no cuidado com o filho.

*A exceção é a mãe que fica. Então, temos que trabalhar muito com ela.* (P2)

*Enquanto o bebê está na UTIN, essa mãe vem visitá-lo esporadicamente. E como vamos fazer para manter sua produção de leite quando esse bebê estiver apto para a via oral, já que ela não fica na unidade? No final, o bebê está pronto para receber alta, mas a amamentação não aconteceu. Temos que ver uma forma de trabalhar com elas na chegada desse bebê na UTIN.* (P12)

*É importante pensar que essas mães estão operadas, andam de ônibus, moram em morros e vão embora com 48 horas, e têm que retornar para passar um dia inteiro no hospital. Elas têm filhos pequenos; têm toda uma questão social.* (P3)

*A mãe voltou para casa. Parece que os afazeres domésticos a afogam. Sem contar que as primeiras 72 horas são cruciais para se fazer um trabalho com essa mãe, ainda dentro da apojadura, criar uma rotina e desmistificar algumas coisas. Apropriar a mãe de seu papel com relação ao seu filho.* (P10)

*A maternagem e paternagem acontecem após o nascimento, e não durante a gestação. Se após o nascimento nós interrompemos esse vínculo, para restabelecer, é muito mais difícil, e isso faz com que os pais fiquem cada vez mais distantes e acabem achando que quem está ali na assistência cuida melhor do filho deles do que eles mesmos.* (P16)

*Isso influencia na alta hospitalar. O fluxo de cuidados de inserir a mãe e o pai no cuidado com seu bebê está acontecendo tardiamente na UTIN.* (P15)

*É preciso considerar que ter a mãe próxima reduz o tempo de internação do RN.* (P12)

Entre as diversas barreiras encontradas, o grupo de trabalho selecionou 11 para serem trabalhadas no projeto de implementação das melhores evidências. Essas barreiras foram escolhidas pela relevância e viabilidade apontadas pelos profissionais do grupo de trabalho. A participação dos profissionais e mães permitiu identificar barreiras em diferentes áreas. Nos meses de debates, as 11 barreiras selecionadas foram ajustadas a partir de estratégias junto aos profissionais de saúde, familiares e gestão, o que possibilitou a implantação do primeiro protocolo de amamentação para prematuros na instituição^(17)^. Cabe ressaltar que o protocolo foi construído baseado nas melhores evidências científicas e seguindo o padrão da instituição para melhoria do cuidado prestado; por isso, seu foco esteve relacionado diretamente à superação das barreiras encontradas, não sendo trabalhados os facilitadores, tópicos em que a equipe já demonstrava êxito.

## DISCUSSÃO

Em relação aos facilitadores e barreiras relacionados à infraestrutura e aos processos intersetoriais, cabe refletir que, apesar de existir uma legislação definindo diretrizes para a organização da atenção integral e humanizada ao recém-nascido internado em UNs, ainda muitos locais no Brasil apresentam limitações na rotina e na estrutura física oferecida aos pais e funcionários^(18)^. Pesquisa realizada em 606 maternidades, de 408 municípios brasileiros, apontou falta de infraestrutura hospitalar e falta de privacidade relacionadas às refeições oferecidas, além de inadequações de estrutura para banho, repouso e falta de roupa de cama^(19)^.

Outras pesquisas também identificaram como barreiras para amamentação de prematuros: infraestrutura deficitária da UN; distância entre a UTIN e o alojamento materno; falta de rotina dos profissionais de saúde; inadequações nos banheiros; mobiliário como cadeiras quebradas e em quantidade insuficiente; ausência de local para banho e para descanso; e ausência de lavanderia para lavagem e secagem das roupas das mães que acompanham seus bebês^(20-22)^.

Em relação à falta de local para extração e armazenamento de leite, é importante pontuar que o BLH do hospital não funciona no período de 24 horas e nos finais de semana, e, por isso, seria importante a existência de um posto de coleta ou local para armazenamento do leite extraído. O BLH tem importante papel no estabelecimento da amamentação dos prematuros durante a sua internação, auxiliando no ganho de peso dos RNPTs que recebem leite humano pasteurizado ou cru, e no apoio às mães para manutenção da lactação e extração do leite materno^(23)^.

Sabe-se que o leite materno apresenta propriedades imunológicas essenciais para auxiliar na maturação gastrointestinal do prematuro, na prevenção de infecções, no melhor desempenho neurocomportamental e no vínculo mãe-filho^(1,7)^. A extração de leite deve começar o mais precoce possível e se manter, no mínimo, oito vezes ao dia em mães que ainda não ofertam o peito, garantindo a manutenção da produção láctea. É fundamental que os profissionais modifiquem suas práticas hospitalares, adotando a extração e oferta precoce de leite materno para os RNPTs^(2,9,10)^.

No que tange aos facilitadores e barreiras relacionados às rotinas e aos cuidados dos profissionais de saúde, pesquisa apontou como facilitadores da amamentação de prematuros orientação e apoio da equipe, capacitação e adequado número de profissionais na UTIN^(20)^. Enquanto isso, as barreiras encontradas foram: falta de incentivo e orientação à mãe; dificuldade na comunicação entre profissionais de saúde e mães; falta de preparo das mães no pré-natal; e falta de humanização do atendimento^(21)^.

A comunicação efetiva é fundamental desde o primeiro contato da mãe com a equipe neonatal. É nela que serão estruturados os processos de confiança e parceria para internações prolongadas, durando de semanas até meses. Cabe ainda pontuar que a comunicação pouco efetiva dentro da equipe e entre os setores gera orientações divergentes, inconsistência nas informações, omissão ou repasse de informações errôneas e registros ilegíveis, influenciando a segurança do paciente^(1,24)^.

Em relação às orientações sobre amamentação, iniciar precocemente ações contínuas de educação durante o pré-natal sobre amamentação fortalece e prepara os membros familiares para a chegada do recém-nascido. Também, é notória a importância da abordagem da gestante ainda no pré-parto e durante a internação na maternidade como decisórios de uma internação mais orientada e tranquila^(1-3,21)^.

As mães reforçam a importância de receberem informação e apoio dos profissionais nos primeiros dias pós-parto quando surgem as dúvidas e dificuldades. Nesse período, ainda estão internadas e cercadas de profissionais capacitados para oferecer esse serviço^(22)^.

Destaca-se a necessidade de formação de grupos de apoio intra-hospitalares e no pós-alta para maior vinculação do binômio, esclarecimento de dúvidas mais frequentes e promoção de altas mais seguras^(1,2)^. O apoio da rede social é fundamental para amamentação exclusiva, sustentando a presença da mãe na unidade, e reduzindo o tempo de internação do RN e a sobrecarga da mulher^(4,25)^.

Os principais motivos relatados pelas mães para o desmame total ou parcial após a alta estão relacionados a questões culturais e educacionais, sendo necessárias orientações quanto à produção láctea e ao processo de amamentação, a fim de reduzir a percepção da baixa produção de leite^(8)^.

Quanto aos facilitadores e barreiras relacionados às mães e familiares, estudo apontou que, de acordo com os profissionais de saúde da UTIN, os facilitadores da amamentação dos prematuros são: presença constante da mãe com acesso livre na unidade; Método Canguru com contato pele a pele; interação e vínculo mãe-bebê; início e estímulo precoce; condições do RN; e desejo da mãe^(20)^. Por outro lado, indisponibilidade da mãe, baixa produção de leite, separação mãe-bebê, questões sociais maternas e tempo prolongado de hospitalização do RNPT foram fatores que dificultaram a amamentação de RNPT^(21)^.

Entre as intervenções que melhoram os resultados da amamentação, incluem-se políticas como Iniciativa Hospital Amigo da Criança e Método Canguru, além da importância da continuidade dos cuidados e apoio em ambientes comunitários e familiares, por meio de visitas domiciliares, com o envolvimento dos pais, das avós e da comunidade^(25)^.

As orientações, o estímulo e apoio que a mulher recebe da família, amigos e profissionais de saúde são fundamentais para ela se sentir acolhida, confortável e capaz de realizar a amamentação^(22)^.

É importante pontuar que a maioria dos desafios da amamentação está relacionada a questões estruturais, não a fatores ou mesmo a decisões pessoais e individuais das mulheres. Garantir a prestação de cuidados respeitosos, culturalmente apropriados, não estigmatizantes e baseados em evidências é fundamental para alcançar a amamentação dos RNPTs e recém-nascidos de baixo peso^(1,2)^.

### Limitações do estudo

Como limitações do estudo, destaca-se a participação de um número restrito de profissionais e mães, o que pode ter limitado a diversidade de perspectivas contempladas. Ressalta-se a necessidade de estabelecer estratégias institucionais que favoreçam maior engajamento e adesão dos diferentes atores às atividades propostas, considerando a relevância dessas ações para o fortalecimento do vínculo das famílias com a equipe de saúde. Além disso, os achados representam as percepções de um conjunto específico de participantes inseridos em uma experiência local, o que restringe a possibilidade de generalização dos resultados para outras instituições e contextos assistenciais.

### Contribuições para a área da enfermagem e saúde

O estudo contribui com a enfermagem, ao possibilitar reflexões sobre as convergências e contrastes relacionados à assistência em amamentação em uma UN. A pesquisa traz como alerta que a manutenção dos cuidados baseados em evidências requer educação permanente dos profissionais de saúde, assim como escuta ativa e estabelecimento de parceria com pais e redes de apoio para o início e manutenção da amamentação de prematuros. Os enfermeiros precisam compreender que os facilitadores e as barreiras refletem a complexidade da implementação de melhores evidências na prática clínica, que perpassa por mudanças culturais, processos de trabalho, relações entre pessoas, envolvendo recursos e interesses da gestão dos serviços.

## CONSIDERAÇÕES FINAIS

Esta pesquisa descreveu a percepção dos profissionais de saúde e mães acerca dos facilitadores e barreiras para implementação de protocolo de amamentação para prematuros, destacando a importância da escuta dos atores envolvidos no processo para implantação das melhores práticas baseadas em evidências e efetivação da amamentação exclusiva de prematuros.

A formação de grupo de trabalho multiprofissional possibilitou que todas as categorias fossem ouvidas, melhorando a comunicação dentro do setor e da instituição. Além disso, a realização de oficinas com as mães permitiu melhor vinculação dessas ao serviço e maior abertura para discussão de problemáticas inerentes à internação dos filhos e aos desafios enfrentados na unidade quanto às orientações e manutenção da amamentação.

A presença de equipe multiprofissional completa e específica da UN e a disponibilidade de serviços que funcionam como pilares na atenção ao prematuro (a exemplo do BLH e seguimento ambulatorial para prematuros), somados à criação de documentos norteadores da prática clínica, foram identificados como os facilitadores para implantação de uma rotina de amamentação. Em contrapartida, as barreiras podem ser representadas pela comunicação deficiente da equipe, inadequações estruturais e de serviços, difícil relacionamento com outros setores do hospital, falta de apoio da gestão e questões que vão além da governabilidade dos profissionais, representadas pela grande vulnerabilidade social do público atendido.

De forma geral, tanto na perspectiva dos profissionais de saúde quanto na das mães, merecem destaque as menções à necessidade de melhoria na infraestrutura do hospital, nas rotinas e processos de trabalho e no comprometimento dos profissionais e familiares.

Ainda permanecem vários desafios a serem superados. No entanto, esta pesquisa possibilitou a identificação de necessidades do serviço, com vistas a melhorar a qualidade do atendimento prestado e promover um cuidado holístico, pautado na atenção à saúde física e emocional dessas famílias.

## Data Availability

Os dados de pesquisa estão disponíveis no corpo do artigo.
